# The VIDAS Data Set: A Spoken Corpus of Migrant and Refugee Spanish Learners

**DOI:** 10.3389/fpsyg.2021.798614

**Published:** 2022-01-20

**Authors:** Margarita Planelles Almeida, Jon Andoni Duñabeitia, Anna Doquin de Saint Preux

**Affiliations:** Centro de Investigación Nebrija en Cognición (CINC), Facultad de Lenguas y Educación, Universidad Nebrija, Madrid, Spain

**Keywords:** language learning, migrants, refugees and asylum seekers, underrepresented learners, language minorities

## Abstract

The VIDAS data set (Verbal Interaction Dataset of Acquired Spanish) presents data from 200 participants from different countries and language backgrounds (50 Philippines with L1 Tagalog; 50 Ukrainians with L1 Ukrainian; 50 Moroccans with L1Arabic; 50 Romanians with L1 Romanian). They completed an oral expression and interaction test in the context of a Spanish certification exam for adult migrants. The aim of the VIDAS data set is to provide researchers in psycholinguistics and second language acquisition with a Spanish spoken corpus of traditionally marginalized and underrepresented learners, providing a compelling data set of oral interactions by migrants and refugees. The corpus contains more than 29 h of recordings of the oral interactions of the participants with trained interviewers, as well as background information about the participants (age, gender, maximum education level, years of residence, and language background). It furthermore contains the scores obtained by the participants in the oral expression and interaction exam. The VIDAS corpus allows for the development of studies on L2 spoken language comprehension and processing, as well as for comparative analyses of language acquisition between different L1 groups at different linguistic levels.

## Introduction

Language data sets and corpora have proven to be crucial in the understanding, modeling and conceptualization of first and second or additional language speech processes, such as acquisition, development, or comprehension (Meurers, 2015; MacWhinney, 2017). Second language acquisition (SLA) research has benefited from data gathered from natural language use in its aim to gain a better understanding of non-native language acquisition and development processes at different linguistic levels (Granger et al., 2015; Myles, 2015). Additionally, spoken data sets of non-native speech are key to training and improving automatic speech recognition (ASR) technologies in the particularly challenging aim of recognizing non-native speech, especially in spontaneous conversational contexts including a diversity of native language backgrounds (Yoon et al., 2010).

In recent decades, the growing interest in the analysis of natural language use both in the field of psycholinguistics and SLA and in the development of ASR has led to the creation of different repositories that collect data by second or additional language learners. While most of them were initially based on written texts, an increasing number of spoken learners’ corpora and data sets are being generated in last years (see Fernández and Davis, 2021, for an overview). Although the dominance of English as the target language is still overwhelming in the field, the growing interest on these resources has led to the development of a growing number of Spanish learners’ speech data sets (see SLABank by MacWhinney, 2017; CORELE by Campillos Llanos, 2014, or SPLLOC by Mitchell et al., 2008). Nevertheless, the samples of speakers mostly comprised by university students and their L1 backgrounds are still rather limited (see McEnery et al., 2019), as well as the speaking tasks that have been used to collect the data. The availability of spontaneously generated samples in conversational contexts of interaction is markedly limited. Consequently, current data sets neglect the representation of a variety of learner profiles with various cultural, academic and language backgrounds, from different learning contexts, and in different task types.

In the current study we aim at providing the community with the VIDAS data set (acronym for Verbal Interaction Dataset of Acquired Spanish). The VIDAS data set presents a database of 200 speech samples of migrant and refugee learners of Spanish in an oral expression and interaction task. The speakers are divided in four groups based on their native languages (L1), thus allowing for comparative analysis of the productions of learners from different linguistic and cultural origins set in the same communicative situation. The VIDAS data set opens an important space for comparative analysis from different perspectives and processes, such as language comprehension and production, and at different levels (phonological, grammatical, lexical, pragmatic, and discursive). Importantly, the VIDAS data set provides speech data of migrants and refugees, representing an opportunity to analyze these processes in traditionally marginalized and underrepresented samples and in conversational settings. Although transcribed and annotated corpora are, undoubtedly, a highly valuable tool for research in SLA,^1^ we believe that access to raw data allows for researchers to approach their analysis from their own paradigms and perspectives, avoiding underlying assumptions in transcription processes that may influence and modify the interpretation of the data depending on the purposes of the study (e.g., Leclercq, 2020).

Applied linguistics, psycholinguistics, and SLA researchers have largely studied the factors involved in the acquisition of an additional language from different perspectives. Nevertheless, non-native language learning in migrants and refugees has not received that much attention, and these populations are still understudied and often ignored or disregarded. After the proposal of a sampling bias in the field of psychology, with research mainly focusing on a very limited participant profile – namely, WEIRD participants; Western, Educated, Industrialized, Rich, and Democratic (Henrich et al., 2010), different researchers in applied linguistics and SLA have raised concerns on the existence of a similar bias also affecting their area of expertise (Bigelow and Tarone, 2004; Ortega, 2005, 2019; Andringa and Godfroid, 2019, 2020). This has led to a call for researchers in this field to go beyond this apparent comfort zone and “demonstrate and make a case for the impact of their work beyond the walls of the academy, in a society that faces many real linguistic needs and questions” (Andringa and Godfroid, 2020, p. 140). After estimation of Plonsky (2016) of 67% of samples in SLA being comprised of university students, Andringa and Godfroid (2020, p. 138) concluded from their recent metadata analysis that participants in applied linguistics research “are truly WEIRD.” This only yields underrepresentation of certain groups in the understanding of language acquisition posing a clear scientific problem, but also presents an ethical dilemma (see Ortega, 2005, 2019; Andringa and Godfroid, 2019, 2020). Our partial aim with the VIDAS data set is to partially compensate this reality and provide an inclusive corpus from underrepresented samples.

For migrants who have just arrived in a country, the challenge of acquiring at least a basic competence in the host language becomes a pressing need to minimally accommodate to their new environment (Doughty and Long, 2008). Migration policies often include specific levels of language proficiency as a legal requirement to acquire citizenship or work access (Hope, 2011). Basic proficiency in the host language has overarching effects on integration at economic, social, and personal levels, and it is a catalyst of economic opportunities and employability (Majhanovich and Deyrich, 2017), of access to social resources, education, and health care, and of social and political participation (Hou and Beiser, 2006; Albarracin et al., 2019). Furthermore, host language proficiency has been shown to have a deep impact beyond economic and social integration, impacting general well-being (Yates, 2011).

Data and reports on international migration [McAuliffe and Khadria, 2019; European Border and Coast Guard Agency (Frontex), 2020] show that migration is a growing phenomenon. Consequently, the acquisition of a language in a migration context, far from being an exceptional or marginal phenomenon, is nowadays conceived as an extended reality to which the society in general, and the scientific community in particular, must respond consciously and with commitment. In this context, research on the acquisition of a second language in migration and refugee contexts is gaining social and scientific interest. The analysis of SLA processes in these contexts undoubtedly poses a series of specific challenges to SLA researchers, given the conditions and peculiarities of the samples (Nieuwboer and van’t Rood, 2016). In a committed and explicit effort to account for language processing of traditionally marginalized and underrepresented learners, the VIDAS data set provides a compelling repository of Spanish oral interaction by migrants and refugees. Specifically, 200 oral interaction samples have been selected, edited, and published divided into 4 speakers groups split by their nationality, which in turn represent different linguistic backgrounds: Philippines (50), Ukrainians (50), Moroccans (50), and Romanians (50).

First and previously known languages have proven to be core factors affecting the acquisition and development of an additional language (see Odlin, 2003; Ringbom, 2007; Jarvis and Pavlenko, 2008 for a review). In this sense, the typological distance between languages and their formal similarity has been widely recognized as influencing factors for the acquisition of additional languages (Cenoz, 2001; Ringbom, 2001). For instance, acquiring the morphosyntactic system and the grammatical and pragmatic uses of articles in Spanish can be especially complex for learners speaking a Slavic language, such as Ukrainian, who do not use articles. This will not, however, pose the same degree of difficulty for speakers of Romanian, a Romance language with many similarities to Spanish. An Arabic speaker, on the other hand, will very likely struggle to recognize and produce minimal phonetic pairs, such as p/b, e/I or o/u, whereas a Filipino speaker will easily perceive, and most likely produce, these contrasts. Hence, the peculiarities, similarities and differences, and the proximity between the linguistic and sociocultural systems of each language call for a case-by-case analysis, attending to each combination of languages individually and specifically. Such an approach will allow us to deepen our understanding of the phenomena involved in the acquisition of an additional language from a scientific perspective. But importantly, this will allow us as a community to accurately define adequate pedagogical approaches based on scientific evidence, especially in the face of the increasing interest in learning Spanish worldwide, and in the face of migratory phenomena.

In addition to L1, length of residence (LOR) in the country of immigration is a main factor traditionally associated with the proficiency level attained by additional language learners (Chiswick and Miller, 2001; van Tubergen and Wierenga, 2011). Similarly, the level of education of the speaker has also been proposed as a predictive factor of L2 attainment, specifically in migration contexts (Chiswick and Miller, 2001; Yilmaz and Schmid, 2015; but see Pérez-Vidal and Juan-Garau, 2011, for a discussion on its potential influence in oral skills development). With this in mind, and in addition to the oral productions, the VIDAS data set also incorporates data for LOR, level of education, as well as the scores obtained in the oral interaction task from the exam that the participants completed. This will be useful for further analysis on the influence of one or a combination of these factors.

The VIDAS data set will allow for the development of studies exploring intercultural competence and the acquisition of pragmatics, interlanguage development at different phonological, lexical, morphological, or syntactic levels, or discourse analysis, among others. VIDAS focuses on Spanish as a continuously growing language and its combinations with different L1 that have been traditionally disregarded and underrepresented in the field of SLA (Ortega, 2019). Thus, the VIDAS data set here presented stands as a significant contribution to and progress for this field, by representing the first corpus of Spanish as a migration language of a similar magnitude and scope.

## Participants

The VIDAS data set compiles a selection of oral interaction samples from 200 Spanish learners in the context of migration. All participants completed a semi-structured oral interview with a trained interviewer in the context of the specific linguistic certification test for immigrant workers in the Community of Madrid, Diploma LETRA^2^ (Baralo Ottonello et al., 2016). All the participants included in the final data set passed the examination with a score of at least five out of 10 (see Table 1 for details). Their ages ranged from 19 to 49 years (*M* = 32.35; SD = 6.8), and their length of regular or irregular residence in Spain ranged from 0 (less than 1 year) to 24 years (*M* = 4.99; SD = 4.68). The participants were divided into four groups according to their nationalities and mother tongues (50 Philippines with L1 Tagalog; 50 Ukrainians with L1 Ukrainian; 50 Moroccans with L1 Arabic; and 50 Romanians with L1 Romanian).

**Table 1:** Characteristics of the samples.

	Morocco	Philippines	Romania	Ukraine
Participants	50 (25)	50 (49)	50 (42)	50 (42)
Age	34.3 (6.32)	34.3 (6.22)	31.3 (7.29)	29.6 (6.33)
LOR	7.91 (5.47)	4.65 (4.21)	5.47 (4.49)	2.12 (2.09)
Level of education	4.2 (1.95)	4.25 (1.96)	4.26 (1.97)	4.26 (1.98)
Score	8.82 (1.59)	7.68 (1.64)	9.35 (1.09)	8.69 (1.48)

Number of participants (and number of females), age, length of residence in years (LOR), level of education and mean score in the oral expression and interaction test (with SD).

## Data Collection

The data were collected in the context of a linguistic certification test for immigrant workers from the Community of Madrid^3^ between the years 2011 and 2016. All the participants first completed a short questionnaire that collected information about their personal data and linguistic history. They then participated in an individual interview with an expert interlocutor structured in three blocks or tasks.^4^ Participants were interviewed individually in a room with two examiners: one of them acted as the main interlocutor, while the other only observed and took notes on the linguistic productions of the interviewees. All interviewers received specific training (a 100-h training course). The interviews were recorded with SONY ICD-PX312 recorders. Informed consent of the participants was collected orally, given the high variability in their levels of reading competence, especially in relation to their level of literacy.^5^

## Data Curation

The edition process of the raw recordings had two steps. First, we deleted the first minutes of the conversations where explicit consent was given by each participant and basic personal information was collected. And second, we silenced all the bits where any specific piece of personal information was given in the middle of the conversation (e.g., family name or address details). This preprocessing of the audios was conducted in Audacity. Following this process, the original raw data consisting of 32 h 53 min 28 s was edited and converted into a data set of 29 h 37 min 43 s. The segments deleted were similar in length for all participants and the correlation between the length of the original and edited audio clips was very high (*r* = 0.96, *p* = <0.001).

## Data Set Overview and Description

**Figure 1:**
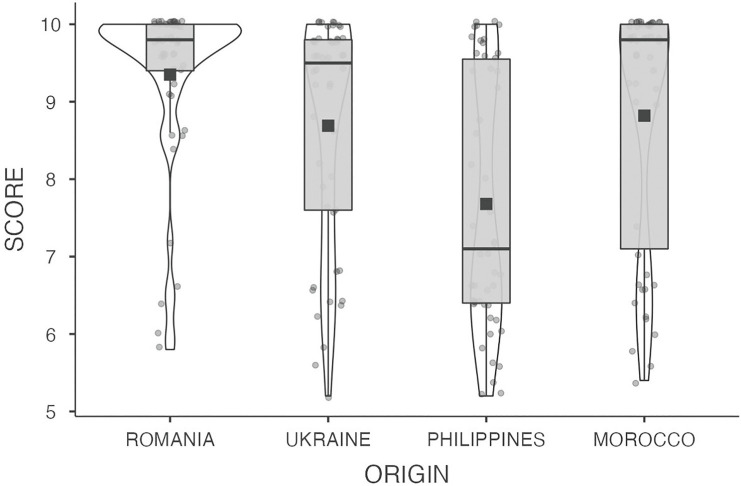
Box plots of the scores obtained by each group of participants in the oral expression and interaction test. The horizontal black lines represent the median score per group, and the black squares correspond to the means.

The VIDAS full data set can be found in https://figshare.com/articles/dataset/THE_VIDAS_DATASET/16578686. It includes, on the one hand, the 200 recordings of the oral interviews of all participants. The audio clips presented in the data set include the whole recording of the interviews. All the slots containing personal information and those bits that could violate the anonymity of the participants were silenced. On the other hand, the data set includes a summary Microsoft Excel® spreadsheet with the linguistic and sociodemographic data corresponding to each participant. The audio files are conveniently labeled with the same code that is presented in the spreadsheet where we provide background information on the participants’ age at the time of data collection (in years), gender, nationality, reported L1(s), and level of education. Level of education was coded as follows: no formal education = 1, primary school = 2, secondary school = 3, high school = 4, professional training = 5, and university = 6. Along with these data, the spreadsheet also presents the score obtained by each of the participants in the oral interaction test. The distributions of the results in the oral examination split by the country of origin of the participants are also presented in Figure 1.

We also present a first approach to the general analysis of the data in which the possible relationship between the scores obtained in the test and the time of residence in the country (LOR) is analyzed, as well as possible differences as a function of the country of origin, being this factor directly associated with participants’ different L1s. Years of residence in the host country and scores obtained in the oral interaction test were found to be moderately positively correlated (*r* = 0.36, *p* < 0.001). In line with other preceding studies, the longer the residence in the country, the higher the level of exposure to Spanish would be, consequently improving the competence in such language. In order to analyze the influence of the country of origin (and therefore their L1) in the scores, we conducted a Kruskal-Wallis test, given that the data were not normally distributed. The results showed a significant difference between the groups (*p* = <0.001). A Dwass-Steel-Critchlow-Fligner test revealed that the Philippine group obtained the lowest scores in the test, and their results differed significantly from those from every other group (all *w*s > 4 and *p*s < 0.004). The Romanian group obtained the highest scores, being similar to those obtained by the Moroccan group but significantly larger than those obtained by the Ukrainian participants (*w* > 3.5 and *p* < 0.01). There were no other significant differences between the scores.

## Conclusion

The VIDAS data set is presented as the first repository of its kind for Spanish as a migration language. It includes a series of edited recordings corresponding to a conversation in the context of an oral interaction test that is part of an official examination (Diploma LETRA). Four groups of 50 persons each are presented, corresponding to 4 different countries of origin and 4 different mother tongues, thus providing an inclusive data set. We believe that this data set will open new avenues of research and analysis in the areas of applied linguistics, SLA and psycholinguistics. The samples and data presented allow for different analysis from various perspectives. Researchers can use this data set, among other things, to explore the influence of different sociodemographic factors on lexical sophistication, interlanguage and development of different grammatical, phonetic, sociopragmatic, or discursive aspects. The recordings obtained in the same contextual situation from different samples representative of four groups with different languages of origin could result in a valuable tool for the development of contrastive analysis with different combinations of native languages that have been traditionally underrepresented in this field. Additionally, access to real L2 speech samples may serve equally the L2 Spanish teaching community – both learners and teachers – in the development of different kind of educational strategies and resources (see, for instance, Fono.ele corpus – reported in Blanco Canales, 2011).^6^

Finally, it is worth noting that the VIDAS data set constitutes a realistic snapshot of Spanish migrant situation. In the data set, one can find from a recent graduate in Medicine from Romania awaiting the validation of her degree to be able to work in Spain after only 8 months of residence in the country (i.e., participant 5_090) to a domestic worker who left her entire family in Philippines and has been living in Spain for 2 years (i.e., participant 5_056); this nicely exemplifies the plethora of individual realities that constitute the regular and irregular immigration reality in Spain, pointing also to different paths in the acquisition of Spanish as an additional language in migration contexts.

## Data Availability Statement

The data sets presented in this study can be found in online repositories. The names of the repository/repositories and accession number(s) can be found in the article/supplementary material.

## Ethics Statement

Ethical review and approval was not required for the study on human participants in accordance with the local legislation and institutional requirements. Written informed consent for participation was not required for this study in accordance with the national legislation and the institutional requirements.

## Author Contributions

MP and JD developed the idea together, analyzed the data, and drafted the manuscript. AD coordinated the data acquisition. All authors approved the final version after discussing the intellectual content and authors agreed to be accountable for all aspects of the work.

## Funding

This study has been partially funded by the Ministry of Science, Innovation, and Universities from the Spanish Government (FFI2017-83166-C2-2-R; PGC2018-097145-B-I00; and RED2018-102615-T), by the Comunidad de Madrid (H2019/HUM5772; and H2019/HUM-5705), and by the Cátedra Global Nebrija-Santander del Español como Lengua de Migrantes y Refugiados.

## Conflict of Interest

The authors declare that the research was conducted in the absence of any commercial or financial relationships that could be construed as a potential conflict of interest.

The handling editor declared a past collaboration with one of the authors JD.

## Publisher’s Note

All claims expressed in this article are solely those of the authors and do not necessarily represent those of their affiliated organizations, or those of the publisher, the editors and the reviewers. Any product that may be evaluated in this article, or claim that may be made by its manufacturer, is not guaranteed or endorsed by the publisher.

## Footnotes

^1^Some of the samples from this data set have already been transcribed and made available as part of a different corpus with more limited purposes (see Nebrija-Inmigra corpus, in SLA-Talkbank, available at https://slabank.talkbank.org/access/Spanish/Nebrija-INMIGRA.html).

^2^ http://www.diplomaletra.com/

^3^The test certifies a level of competence in Spanish that exceeds level A1, but with specific content which does not correspond to the A2 level, taking into account the concept of partial competence in a language, and assuming that the development of the different skills in immigrant workers is irregular (see Baralo Ottonello et al., 2016).

^4^ https://figshare.com/articles/dataset/THE_VIDAS_DATASET/16578686

^5^At the beginning of the recording, the participants were asked for their express consent for the audio recording of the test, and it was explained to them that the data would be used for research purposes. Interviewers were instructed to adapt their discourse to the level of competence in the language of each participant.

^6^Available at: http://www3.uah.es/fonoele/
